# Transcriptome Analysis Reveals an Inhibitory Effect of Dihydrotestosterone-Treated 2D- and 3D-Cultured Dermal Papilla Cells on Hair Follicle Growth

**DOI:** 10.3389/fcell.2021.724310

**Published:** 2021-09-17

**Authors:** Yufan Zhang, Junfei Huang, Danlan Fu, Zhen Liu, Hailin Wang, Jin Wang, Qian Qu, Kaitao Li, Zhexiang Fan, Zhiqi Hu, Yong Miao

**Affiliations:** Department of Plastic and Aesthetic Surgery, Nanfang Hospital of Southern Medical University, Guangzhou, China

**Keywords:** RNA-seq, 3D model, dermal papilla cells, dihydrotestosterone, androgenic alopecia, chemokines

## Abstract

Dermal papillae are a target of androgen action in patients with androgenic alopecia, where androgen acts on the epidermis of hair follicles in a paracrine manner. To mimic the complexity of the dermal papilla microenvironment, a better culture model of human dermal papilla cells (DPCs) is needed. Therefore, we evaluated the inhibitory effect of dihydrotestosterone (DHT)-treated two-dimensional (2D)- and 3D-cultured DPCs on hair follicle growth. 2D- and 3D-cultured DPC proliferation was inhibited after co-culturing with outer root sheath (ORS) cells under DHT treatment. Moreover, gene expression levels of β-catenin and neural cell adhesion molecules were significantly decreased and those of cleaved caspase-3 significantly increased in 2D- and 3D-cultured DPCs with increasing DHT concentrations. ORS cell proliferation also significantly increased after co-culturing in the control-3D model compared with the control-2D model. Ki67 downregulation and cleaved caspase-3 upregulation in DHT-treated 2D and 3D groups significantly inhibited ORS cell proliferation. Sequencing showed an increase in the expression of genes related to extracellular matrix synthesis in the 3D model group. Additionally, the top 10 hub genes were identified, and the expression of nine chemokine-related genes in DHT-treated DPCs was found to be significantly increased. We also identified the interactions between transcription factor (TF) genes and microRNAs (miRNAs) with hub genes and the TF–miRNA coregulatory network. Overall, the findings indicate that 3D-cultured DPCs are more representative of *in vivo* conditions than 2D-cultured DPCs and contribute to our understanding of the molecular mechanisms underlying androgen-induced alopecia.

## Introduction

Hair follicle development and growth depends on reciprocal epithelial–mesenchymal interactions, and the starting point of the initial signals is thought to originate from the mesenchymal dermis (Dhouailly, 1973). The dermal papillae, a cluster of mesenchymal cells at the base of the hair follicle, are considered to play an important role in the hair cycle by regulating the growth and activity of various cells in hair follicles through the secretion of diffusible proteins to the epidermis of hair follicles (Andl et al., 2002).

Androgen alopecia (AGA), also known as male hair loss, affects 50% of men worldwide and is the most common form of hair loss in men (McElwee and Shapiro, 2012). AGA is characterized by the gradual miniaturization of hair follicles and a premature transition from anagen to catagen induced by androgens (Jahoda, 1998). The dermal papilla cells (DPCs) of bald scalp contain more 5-α-reductase, leading to the conversion of peripheral testosterone into a more active dihydrotestosterone (DHT), which has an affinity that is five times as strong as that of testosterone. DHT binds to androgen receptors (ARs) in the nucleus, initiating a cascade of reactions and triggering this effect (Dallob et al., 1994). Overall, DPCs are the targets of androgen action in AGA hair follicles. Androgen drives DPCs to act on themselves in a paracrine manner in hair follicle epithelial cells, resulting in the miniaturization of hair follicles and changes in the hair follicle cycle (Randall et al., 2001). Thus, altering the expression of DPC-related genes under DHT treatment may be a key factor in androgen-potentiated balding.

To elucidate the regulatory effect of DHT-treated DPCs on epidermal cells of hair follicles, a co-culture model was established in 1995 in which outer root sheath (ORS) cells were cultured in the upper compartment and DPCs in the lower compartment of Transwell culture dishes (Itami et al., 1995). Subsequently, several scientists have used this co-culture model to explore the mechanism of AGA. Kwack et al. (2008) found that DHT-mediated Dickkopf Wnt signaling pathway inhibitor 1 (DKK1), secreted by DPCs, is involved in DHT-driven balding. Kitagawa et al. (2009) observed that DHT inhibited the proliferation of keratinocytes, which was induced by Wnt3a through Wnt/β-catenin signaling. Leirós et al. (2017) reported that DHT activates GSK-3β in DPCs, inhibiting the differentiation of hair follicle stem cells by phosphorylating β-catenin (Kitagawa et al., 2009); they also found that DHT could downregulate the expression of Wnt5a and Wnt10b by stimulating DPCs from patients with AGA. Inui et al. (2002) examined the role of androgen-induced transforming growth factor-β (TGF-β1) derived from DPCs in AGA, which is involved in the growth inhibition of epithelial cells in a co-culture system. Kwack et al. (2012) reported that DHT-inducible IL-6 inhibits the proliferation of human follicular keratinocytes. The above findings were obtained using the co-culture model of DPCs and hair follicle epidermal cells.

In previous studies, the DPCs were cultured in two-dimensional (2D) monolayers of co-culture systems; however, DPCs *in vivo* are surrounded by an extracellular matrix (ECM), clustered into a special 3D spherical and structured dermal papilla. Therefore, the 2D model has some limitations because it is unable to mimic the complexity of the hair follicle microenvironment. Compared with 2D culture conditions, a significant advantage of 3D culture is that it reduces the differences between cell models *in vitro* and *in vivo*. Additionally, 3D cultured cells are more representative of the complexity of *in vivo* conditions (Thippabhotla et al., 2019). With respect to DPCs in particular, previous studies have reported that 3D-cultured DPCs possess a greater hair follicle regenerating ability than 2D-cultured DPCs (Lin G. et al., 2020), indicating considerable differences in 2D- and 3D-cultured DPCs. Hair inductivity sharply decreases when DPCs are expanded in 2D culture because of the absence of the 3D microenvironment (Ohyama et al., 2012; Abaci et al., 2018). Several different approaches have been employed to construct DP spheroids in an attempt to restore the inductive characteristics of DPCs, such as hanging drop culture (Lin et al., 2016), low adhesion biomaterial surfaces (Osada et al., 2007), and hydrogel culture (Tan et al., 2019). In this study, we used a low adhesion biomaterial surface approach to culture DPCs in 3D and speculated that DHT-induced 2D- and 3D-cultured DPCs may secrete different kinds of proteins, inhibiting the proliferation of hair follicle epidermal cells by activating different pathways.

RNA sequencing (RNA-seq) is a relatively new method for analyzing eukaryotic transcripts, and it is cheaper and more effective for identifying previously unknown gene signatures compared with microarray and Sanger sequencing technology (Nagalakshmi et al., 2008). In the present study, we used RNA-seq to identify differentially expressed genes (DEGs) in 2D- and 3D-cultured DPCs under DHT treatment.

## Materials and Methods

### *In vitro* Dermal Papilla Cells Cultured in 2D

Human hair follicles were taken from the balding (frontal) area of male patients undergoing hair transplantation surgery. Ethical approval and informed consent were obtained preoperatively from Nanfang Hospital of Southern Medical University. Dermal papillae were isolated from the bulbs of hair follicles, plated in a cell culture flask (Corning Inc., NY, United States) and cultured in Dulbecco’s Modified Eagle Medium (DMEM; Gibco, MA, United States) supplemented with 1% (v/v) penicillin–streptomycin and 20% (v/v) fetal bovine serum (FBS; Gibco) at 37°C and 5% CO_2_. Once the outgrowth reached 80% confluence, human DPCs were harvested by incubating with 0.25% (w/v) trypsin/EDTA (Gibco) and transferring to new culture dishes at a split ratio of 1:2.

### *In vitro* Outer Root Sheath Cells Cultured in 2D

Outer root sheath cells were isolated from the same hair specimens mentioned above. The hair follicle bulb was separated using microscissors and treated with 0.1% dispase (Invitrogen, CA, United States) for 45 min. The dermal sheaths were separated and removed under a stereoscope, and the epidermis was treated with 0.05% trypsin (Gibco) for 10 min. After terminating digestion, the samples were filtered through a 70-μm strainer (Corning Inc.) and cultured with defined keratinocyte-SFM (KSFM; Gibco) in flasks that were pre-coated with 10 μg/mL human fibronectin (Sigma-Aldrich, MO, United States).

### *In vitro* Dermal Papilla Cells Cultured in 3D

For 3D culture, DPCs were seeded in an ultra-low attachment 96-well plate (Corning Inc.) with DMEM, supplemented with 1% (v/v) penicillin–streptomycin and 10% (v/v) FBS. Spheroids were formed 1 day after seeding and were used in the further assays.

### MTT Assay

Two-dimensional- and 3D-cultured DPCs (8 × 10^3^ cells per well) were seeded into 96-well plates and cultured for two days with DHT at different concentrations. MTT (20 μL; 5 mg/mL) was added to each well, and the cells were incubated for 4 h at 37°C; subsequently 200 μL of dimethyl sulfoxide was added to dissolve the formazan after removing the supernatant products. Proliferation of DPCs was determined spectrophotometrically at 570 nm using an ELISA reader (TECAN Infinite F50, Männedorf, Switzerland).

### Co-culture of Dermal Papilla Cells and Outer Root Sheath Cells

Two-dimensional-cultured DPCs were harvested by incubation with 0.25% (w/v) trypsin/EDTA from the cell culture flask and plated at a density of 1 × 10^4^ cells/cm^2^ in the upper compartment of Transwell culture dishes (Corning Inc.). Dermal papillae spheres of the same quantity as the 2D-cultured cells were also transferred to the upper compartment of another Transwell culture dish. ORS cells (4 × 10^3^ cells/well), which were cultured in FBS-free DMEM on the day before co-culture, were added to the lower compartment of each dish and co-cultured with 2D- and 3D-cultured DPCs. After 1 day, the medium was changed and 10 μM DHT was added to the co-culture system. The number of ORS cells was counted after 3 days.

### Western Blotting Analysis

Protein extracts were isolated from DPCs using a RIPA protein lysis buffer containing a protease inhibitor cocktail (Roche, Switzerland). Protein concentrations were determined using a BCA protein assay kit (Pierce, IL, United States), measuring the absorbance at 562 nm. Total protein (20 μg) was subjected to SDS-PAGE, and the separated proteins were transferred to a polyvinylidene fluoride membrane (Millipore, MA, United States). The membrane was blocked in 5% bovine serum albumin (BSA) for 1 h and probed with appropriate primary antibodies overnight at 4°C. Primary antibodies against the following proteins were used: AR (1:2,000; ab133273, Abcam, Cambridge, United Kingdom), neural cell adhesion molecule (NCAM; 1:2,000, ab75813, Abcam), β-catenin (1:2,000; ab32572, Abcam), Ki67 (1:2,000; ab16667, Abcam), cleaved caspase-3 (1:2,000; ab32042, Abcam), β-actin (1:5,000; ab8226, Abcam), and alpha-tubulin (1:5,000; AC007, ABclonal, MA, United States). After washing with Tris Buffered saline Tween, the blots were incubated with the corresponding secondary antibody (1:1,000; ab150077, Abcam) for 1 h at 20–25°C and photographed using an Odyssey infrared fluorescent scanning imager (Bio-Rad, CA, United States).

### Immunofluorescence

Dermal papilla cells were washed once with PBS and fixed in 4% paraformaldehyde for 20 min at approximately 23°C. Then, the samples were rinsed with PBS three times, permeated with 0.3% Triton X-100 (Solarbio, Beijing, China), and blocked with 3% BSA (Solarbio). Subsequently, the samples were stained with primary antibodies against AR (1:200; Abcam) at 4°C overnight. The next day, DPCs were incubated with Alexa Fluor-568 conjugated anti-rabbit secondary antibody (1:200; Abcam) and 2-(4-amidinophenyl)-6-indolecarbamidine dihydrochloride (DAPI; 1:200; Abcam) for 1 h at 20–25°C. Finally, the samples were imaged using a fluorescence microscope (IX71 FL; Olympus, Tokyo, Japan).

### RNA Extraction, Library Construction, and Sequencing

For RNA-seq analysis, 2D- and 3D-cultured DPCs were treated with or without DHT for 3 days. Samples were collected from 2D and 3D treated and control groups, and total RNA was extracted using a TRIzol kit (Invitrogen), according to the manufacturer’s instructions. RNA quality was evaluated using Agilent 2100 Bioanalyzer (Agilent Technologies, CA, United States) and examined using RNase-free agarose gel electrophoresis. After total RNA extraction, eukaryotic mRNAs were enriched using oligomeric (dT) beads, and prokaryotic mRNAs were enriched by removing rRNAs using the Ribo-Zero^TM^ magnetic kit (Epicenter, WI, United States). The enriched mRNAs were then fragmented into short sequences using fragmentation buffer and reverse transcribed into cDNA using random primers. Second strand cDNA was synthesized using DNA polymerase I, RNase H, dNTP, and buffer. The QiaQuick PCR extraction kit (Qiagen, Venlo, Holland) was used to purify the cDNA fragments, repair base ends, and connect the Illumina sequencing adapters. The sizes of the ligated products were determined using agarose gel electrophoresis. The products were amplified by using PCR and sequenced using the Illumina NovaSeq 6000 by Gene Denovo Biotechnology Co. (Guangzhou, China). Gene expression analysis of identified transcripts was performed using the DESeq2 package in R software. Genes with changes in expression of *p* < 0.05 and log2| FC| > 1 were considered to be DEGs.

### Correlation Analysis of Replicates

Correlation analysis of the three parallel experiments was performed using R software^1^ to evaluate the reliability and operational stability of the experimental results. The repeatability was evaluated by calculating the correlation coefficient among the three replicates: the closer the correlation coefficient to 1, the stronger the repeatability among the three parallel experiments.

### Principal Component Analysis

Principal component analysis (PCA) was performed using an omicshare tool.^2^ We performed PCA to reveal the structure or relationship of the samples.

### Identification of Differentially Expressed Genes

RNA differential expression analysis of the four groups (DHT-2D, control-2D, DHT-3D, and control-3D) was performed using DESeq2 (Love et al., 2014) software. Genes/transcripts with *p*-value [false discovery rate (FDR)] < 0.05, and logarithmic fold change value (log2FC) > 1 were considered to be DEGs. Overlapping genes between two comparison groups (DHT-2D vs. control-2D and DHT-3D vs. control-3D) were presented using Venn diagrams. DEGs were annotated using Gene Ontology (GO) and the Kyoto Encyclopedia of Genes and Genomes (KEGG) databases.

### Gene Ontology and Kyoto Encyclopedia of Genes and Genomes Enrichment Analysis

To further identify section “Advantages of Control-3D Over Control-2D in Simulating Dermal Papilla Cells *in vivo*,” we used KEGG^3^ to specify relevant molecular processes of control-2D and control-3D. Subsequently, KEGG and GO were performed using The Database for Annotation, Visualization, and Integrated Discovery (DAVID; version 6.8^4^) to analyze the associated cellular pathways. Specifically, biological classification and molecular function enrichment were assessed for the DEGs in the control-2D vs. DHT-2D, and control-3D vs. DHT-3D, as well as overlapping DEGs between the two comparison groups (Huang et al., 2007).

### Gene Set Enrichment Analysis

To determine statistically significant gene sets among the four groups, the genes were analyzed using Gene Set Enrichment Analysis (GSEA) software v3.0, available from the Broad Institute.^5^ The dataset was input with the annotation file “hallmark gene sets.” Enriched gene sets were identified based on *p* < 0.05 and FDR *q* < 0.25.

### Construction of a Protein–Protein Interaction Network and Analysis of Module

The Search Tool for the Retrieval of Interacting Genes (STRING^6^) is an online database for analyzing protein–protein interaction (PPI) networks of genes (Szklarczyk et al., 2017), which determines genes as nodes and interactions as lines in a network. In the present study, the STRING database was used to construct the PPI network of overlapping DEGs between DHT-2D vs. control-2D and DHT-3D vs. control-3D. Networks with a combined score >0.4 were defined as statistically significant interactions. Cytoscape (v3.7.1) software was used to visualize the network file and present core and hub gene biological interactions. The plugin Molecular Complex Detection (MCODE) (version 1.4.2) of Cytoscape is an application for clustering a given network based on topology to find densely connected regions. The PPI networks were depicted using Cytoscape, and the most significant module in the PPI networks was identified using MCODE. The standard for selection was as follows: MCODE scores >5, degree cut-off = 2, node score cut-off = 0.2, max depth = 100, and node *k*-score = 2.

### Hierarchical Clustering

Unsupervised hierarchical cluster analysis was performed on the ECM–receptor related genes, reported AGA-related genes, and overlapping DEGs in the two comparison groups (control-2D vs. DHT-2D and control-3D vs. DHT-3D). Raw *z*-scores were first calculated from counts of the four groups and then subjected to agglomerative hierarchical clustering analysis based on Ward’s method and Euclidean distance. Bioinformatics analysis was performed, and heat maps were generated using an omicshare program (see text footnote 2).

### Hub Genes Selection and Analysis

Hub genes were obtained from an application plugin in Cytoscape called CytoHubba. After constructing the network of genes, the top 10 genes with degree ≥10 were identified as hub genes. The Biological Networks Gene Oncology tool (BiNGO) (version 3.0.3), another APP plugin in Cytoscape, calculates overrepresented GO terms by GO_full analysis in the network and displays them as a network of significant GO terms. The term “GO_full analysis” is an option in the BiNGO settings that includes biological processes, cellular components, and molecular functions.

### Quantitative Real-Time Reverse Transcription Polymerase Chain Reaction

Total RNA was extracted using a TRIzol reagent (Invitrogen) to evaluate expression levels of the 10 hub genes. cDNA was reverse transcribed from 2 mg of RNA using the SYBR Prime-Script RT-PCR Kit (TaKaRa Bio, Japan). Quantitative real-time reverse transcription polymerase chain reaction (qRT-PCR) was performed using the ABI Prism 7900HT Sequence Detection System (Life Technologies, CA, United States), according to the manufacturer’s protocol. Relative gene expression levels were calculated using the 2^–ΔΔ*Ct*^ method.

### Transcription Factor-Gene and MicroRNA Interactions With Hub Genes and Transcription Factor–MicroRNA Coregulatory Network

The NetworkAnalyst^7^ platform was used to identify transcription factor (TF)-gene and microRNA (miRNA) interactions with hub genes and the TF–miRNA coregulatory network.

### Statistical Analysis

Statistical analysis was conducted using one-way ANOVA and two-tailed Student’s *t*-test in GraphPad Prism 8 (GraphPad Software Inc., CA, United States). Results are presented as the mean ± standard deviation (SD), and statistical significance was set at *p* ≤ 0.05. Heat maps were used to display the hierarchical groupings of the gene expression profiles of the samples using R package. Each experiment was repeated at least three times.

## Results

### Selection of Dermal Papilla Cells With Relatively High Expression of Androgen Receptor for Co-Culture Model

**FIGURE 1:**
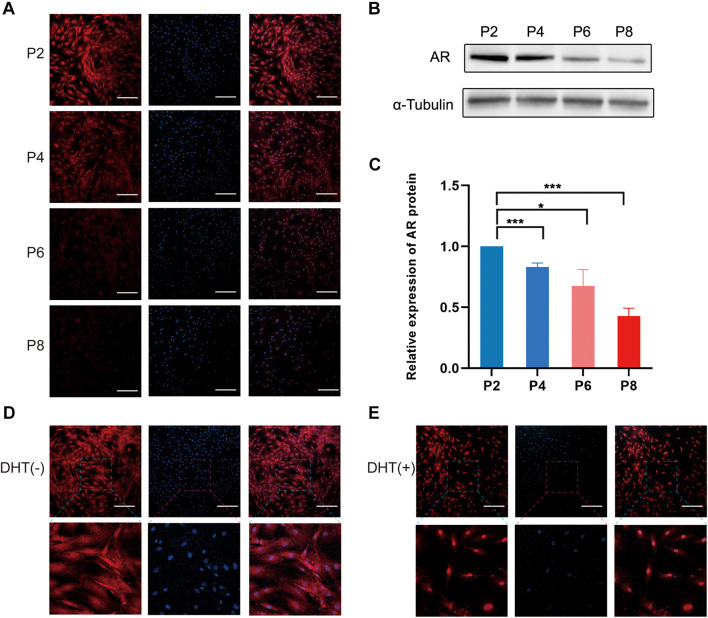
Expression of androgen receptor in cultured DPCs and AR translocation from the cytoplasm to nucleus following DHT treatment. **(A)** Immunofluorescence images of AR expression of DPCs in different passages. Scale bar = 200 μm. **(B)** Western blotting analysis of AR in different passages. **(C)** Relative expression of AR protein in P2, P4, P6, and P8. Immunofluorescence images of AR expression **(D)** in the cytoplasm in the absence of DHT, and **(E)** in the nucleus in the presence of DHT. Results are presented as mean ± SD. *p < 0.05; ***p < 0.001, compared with P2 group by one-way ANOVA and Student’s *t*-test. AR, androgen receptor; DHT, dihydrotestosterone; DPC, dermal papilla cells.

The results of immunofluorescence staining of DPCs at passage (P)2, P4, P6, and P8 showed that the AR protein was significantly expressed in P2 and P4 compared with that in P6 and P8 (Figure 1A). Similarly, western blotting analysis showed that the AR protein of the DPCs decreased during passage (Figures 1B,C), and the differences between the P2 group and the other groups were statistically significant. Additionally, when DPCs were treated with 10 μm DHT, immunofluorescence staining revealed that the AR had been transferred from the original cytoplasm to the nucleus (Figures 1D,E). We selected P2 DPCs with relatively high AR expression for the co-culture model.

### Establishment of 2D and 3D Dermal Papilla Cell Co-Culture Models With Outer Root Sheath Cells for Androgen Alopecia

**FIGURE 2:**
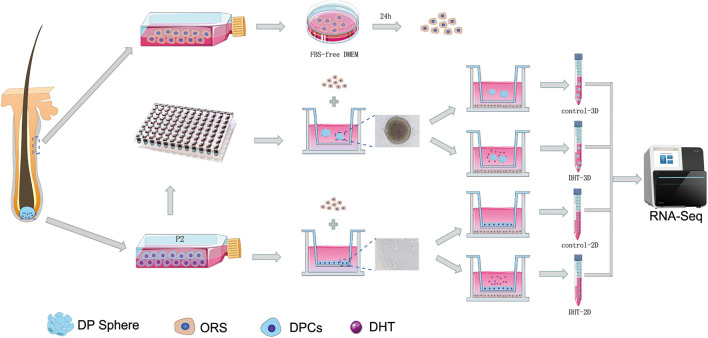
Construction process for the 2D and 3D co-cultured models. The schematic shows the construction process of the 2D and 3D co-culture models containing DPCs and ORS cells. The specific steps are mentioned in section “Materials and Methods.” DPCs and ORS cells were seeded, respectively, in the upper and lower compartments of Transwell culture dishes, with or without of DHT (10 μM). DHT, dihydrotestosterone; DPC, dermal papilla cell; ORS, outer root sheath.

**FIGURE 3:**
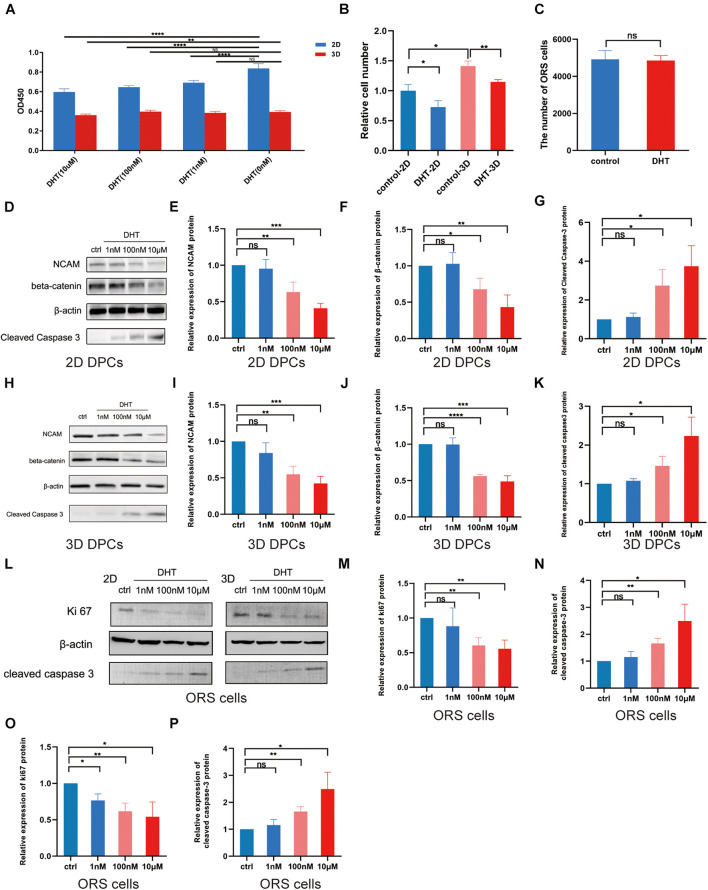
Effect of dihydrotestosterone on outer root sheath cells regulated by dermal papilla cells in 2D and 3D co-culture models. **(A)** Effect of different concentrations of DHT on the proliferation of 2D- and 3D-cultured DPCs. **(B)** Relative ORS cell number in the four groups (control-2D, DHT-2D, control-3D, and DHT-3D). **(C)** Quantity of ORS cells cultured alone in the absence or presence of DHT (10 μM). Western blotting analysis of NCAM, β–catenin, and cleaved caspase-3 in the 2D- **(D)** and 3D-cultured **(H)** DPCs at 0 nM (control), 1 nM, 100 nM, and 10 μM DHT **(D)**. Relative expression of NCAM, β–catenin **(F)**, and cleaved caspase-3 in 2D **(E–G)** and 3D **(I–K)** DPCs. **(L)** Western blotting analysis of Ki67 and cleaved caspase-3 in 2D- and 3D-co-cultured ORS cells at 0 nM (control), 1 nM, 100 nM, and 10 μM DHT. Relative expression of Ki67 and cleaved caspase-3 in 2D **(M,N)** and 3D **(O,P)** ORS cells. The results were obtained using three replicates and presented as mean ± SD. ns, not significant; *p < 0.05; **p < 0.01; ***p < 0.001; and ****p < 0.0001 compared with the respective controls by one-way ANOVA and Student’s *t*-test. DHT, dihydrotestosterone; DPC, dermal papilla cell; NCAM, neural cell adhesion molecule; ORS, outer root sheath.

The construction process of 2D and 3D co-culture models is shown in Figure 2. DPCs are located within a complex ECM environment that involves wide-ranging intermolecular interactions. The 3D co-culture model thus offers a way to simulate the *in vivo* morphology and normal physiological function of these cells. DHT inhibited the proliferation of 2D-cultured DPCs in a dose-dependent manner after 2 days of treatment (*p* < 0.0001); however, only 10 μm DHT significantly inhibited the proliferation of 3D-cultured DPCs (Figure 3A). Additionally, there was a significant decrease in NCAM and β-catenin expression and a significant increase in cleaved caspase-3 expression in the 2D- (Figures 3D–G) and 3D-cultured DPCs (Figures 3H–K) with increasing DHT concentration. The number of ORS cells in the 3D co-culture model was nearly 1.5-times that of the 2D co-culture model. However, there was a decrease in the quantity of ORS cells in both the 2D and 3D co-culture models with an increase in DHT concentration (Figure 3B). DHT did not have an inhibitory effect on ORS cells that were not co-cultured with DPCs, suggesting that the negative effect of DHT on ORS cells (Figure 3C) was mediated indirectly by DPCs. There was a significant decrease in Ki67 (Figures 3L–N) and cleaved caspase-3 (Figures 3L,O,P) expression in ORS cells with increasing DHT concentration.

### Clear Separation of Transcriptional Signatures of Inter-Groups and High Reproducibility of Intra-Groups

**FIGURE 4:**
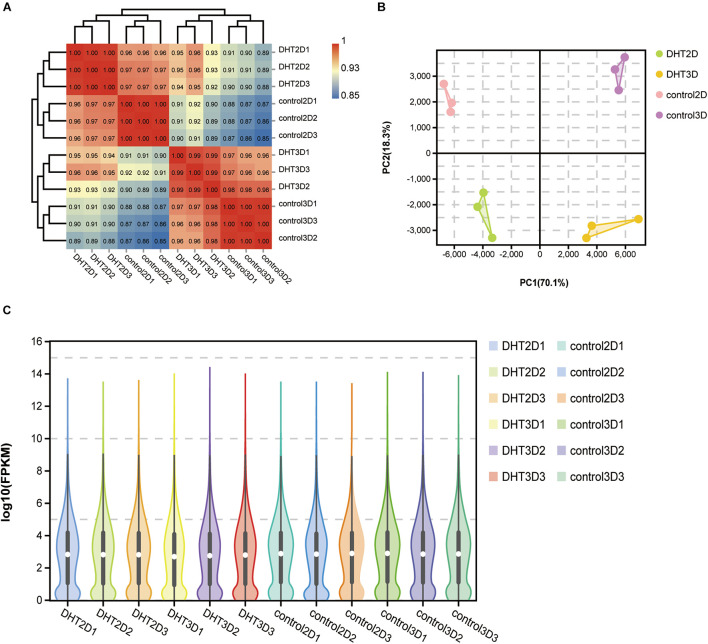
Correlation analysis between samples. **(A)** Logarithm-transformed counts from RNA-seq dataset of control-2D, DHT-2D, control-3D, and DHT-3D used for Pearson’s correlation analysis. *R*^2^ values from Pearson’s correlation analysis were plotted inside the grids of the heatmap. **(B)** PCA analysis. Percentages in PCA axis indicate the proportional variance explained by each PC. Control-2D samples are labeled pink, DHT-2D samples are labeled green, control-3D samples are labeled purple, and DHT-3D samples are labeled yellow, representative of conditions located in the upper right part of plot. **(C)** Violin plot visualizing data density at any location of all genes in each sample, indicating gene abundance expression. White dots represent the median, the black rectangle represents the lower quartile (Q1) range to the upper quartile (Q3), and black lines running through the violin chart represent the confidence interval. The outer shape of the black rectangle is the kernel density estimation, the length of the longitudinal axis of the figure represents the degree of gene dispersion, and the length of the horizontal axis represents the amount of gene expression distribution in a certain ordinate position. DHT, dihydrotestosterone; PCA, principal component analysis.

High-throughput sequencing technology was used to investigate the mRNA profiles of genes in the control-2D, DHT-2D, control-3D, and DHT-3D groups. A total of 12 DPC samples were collected from the models, with three samples from each group. Furthermore, Pearson’s correlation analysis was performed to examine the similarity and discrepancy between the four groups using the normalized fragment counts as the distance between genes. The results showed that samples in the same group were highly similar, while samples from different groups were distinct. The correlation coefficients of the four groups ranged 0.85–0.88 for control-2D and control-3D, 0.96–0.97 for control-2D and DHT-2D, and 0.96–0.98 for control-3D and DHT-3D samples (Figure 4A). PCA results showed that 88.4% of gene expression variation (PC1: 70.1%, PC2: 18.3%) could be explained by the first two PCs (Figure 4B). Two of the four groups in the PCA projection showed a significant distance in the PC space, indicating that the significant variations in gene expression of the different treatment groups can be attributed to the DHT treatment and cell morphological changes in 2D- and 3D-cultured DPCs. A short distance in the intra-group analysis prompted minute variations and high biological replication among the three samples within one group. To directly observe gene abundance at any location in each sample, we used a violin plot to display the degree of dispersion of gene expression (*y*-axis) and data distribution of a vertical coordinate position (*x*-axis). We found that the interquartile distance, median, and gene expression distribution in the intra-group were nearly the same, indicating that the parallel samples had high reproducibility (Figure 4C). The correlation heatmap, PCA analysis, and violin plot demonstrated a clear separation of transcriptional signatures between the four groups and confirmed high-quality RNA-seq data for further functional analysis.

### Advantages of Control-3D Over Control-2D in Simulating Dermal Papilla Cells *in vivo*

**FIGURE 5:**
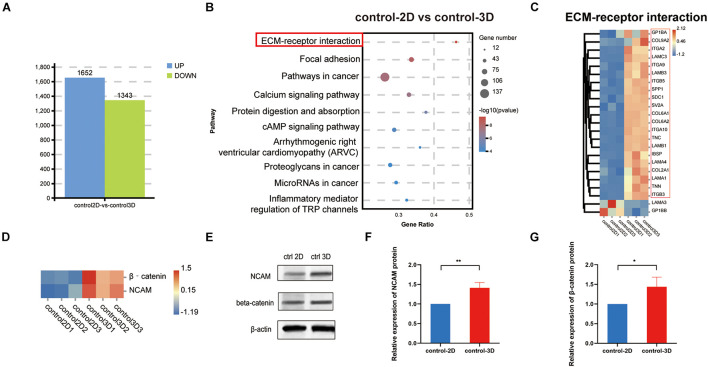
Identification and comparison of 2D and 3D co-culture models in the absence of dihydrotestosterone. **(A)** Quantity of DEGs in control-2D vs. control-3D groups, displayed as a bar chart. Blue represents upregulation and green represents downregulation. **(B)** KEGG analysis of DEGs between control-2D and control-3D. The size of the bubble represents gene number, color depth represents the *p*-value, and the rich ratio represents the gene number/total gene number on the *y*-axis. **(C)** DEGs (22) in the control-2D and control-3D related to the ECM–receptor interaction are depicted as a heatmap. Red indicates upregulation and blue indicates downregulation. **(D)** Expression of β–catenin and NCAM of control-2D and control-3D depicted as a heatmap. Red indicates upregulation and blue indicates downregulation. **(E)** Western blotting analysis of expression of NCAM and β–catenin and the relative expression of NCAM **(F)** and β–catenin **(G)** in control-2D and control-3D DPCs. DEGs, differentially expressed genes; DPC, dermal papilla cell; ECM, extracellular matrix; NCAM, neural cell adhesion molecule. *p < 0.05; **p < 0.01.

To demonstrate that the 3D model is more representative of the *in vivo* situation than the 2D model. The results of the analysis showed that there were 1,652 upregulated genes and 1,343 downregulated genes in the control-2D vs. control-3D group (Figure 5A). KEGG analysis of the DEGs indicates that the DEGs were mainly enriched in ECM–receptor interaction, with the gene number/total gene number (gene ratio) close to 0.5. Among 22 genes enriched in ECM–receptor interaction, unsupervised hierarchical clustering showed that 20 were upregulated and two were downregulated (Figure 5B), suggesting that the ECM was more abundant in the control-3D group (Figure 5C). Additionally, β-catenin expression was significantly higher in the control-3D than that in the control-2D group (Figure 5D), indicating the involvement of the canonical Wnt/β−catenin pathway, owing to 3D aggregation of DPCs. The expression of NCAM and β-catenin was confirmed by western blotting analysis (Figures 5E–G). The upregulated expression of NCAM in the 3D-control showed that the 3D model could exhibit, to some extent, the characteristics of hair follicle development *in vivo*.

### Identification of Differentially Expressed Genes in Two Comparison Groups and Reliability of the Models

**FIGURE 6:**
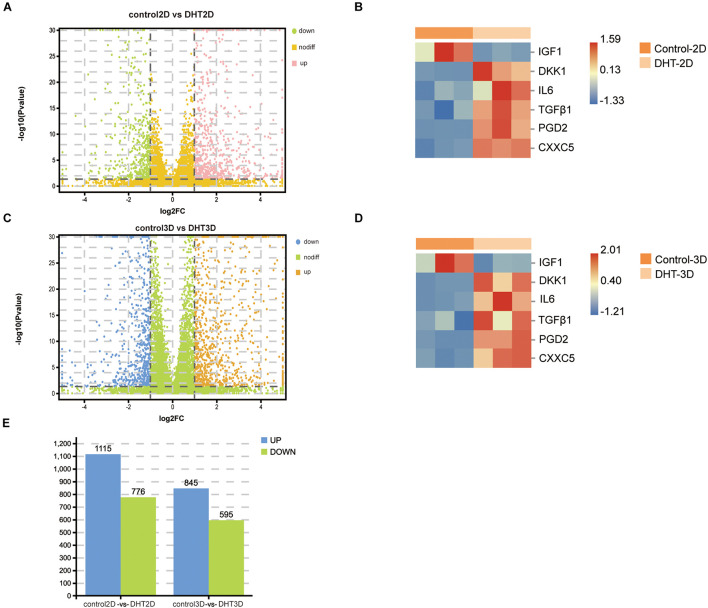
Identification of differentially expressed genes in control-2D vs. dihydrotestosterone-2D and control-3D vs. dihydrotestosterone-3D, and the expression of proven genes. Volcano plot used to determine the DEGs in control-2D vs. DHT-2D **(A)** and control-3D vs. DHT-3D **(C)** using the following criteria: *p* < 0.05 and log2| FC| > 1. Heatmap depicting the expression of six genes in control-2D vs. DHT-2D **(B)** and control-3D vs. DHT-3D **(D)**. Red indicates upregulation and blue indicates downregulation. **(E)** Quantity of DEGs in control-2D vs. DHT-2D and control-3D vs. DHT-3D. Blue represents upregulation and green represents downregulation. DEGs, differentially expressed genes; DHT, dihydrotestosterone.

To further characterize DEGs in the control-2D vs. DHT-2D and control-3D vs. DHT-3D groups, gene expression analysis was conducted on the transcripts (19,727 identified genes) from the two comparison groups (Figures 6A,C). A total of 3,331 DEGs were identified (1,891 in control-2D vs. DHT-2D and 1,440 in control-3D vs. DHT-3D), among which 1,175 were upregulated and 776 were downregulated in control-2D vs. DHT-2D samples, while 845 were upregulated and 595 were downregulated in control-3D vs. DHT-3D samples (Figure 6E). To verify that the model can accurately reflect the effect of DPCs on ORS under androgen stimulation, an unsupervised hierarchical clustering was conducted using six genes (*IGF-1*, *DKK1*, *IL-6*, *TGF*-β*1*, *PTGDS*, and *CXXC5*) (Figures 6B,D). A hierarchical heatmap indicated that IGF-1 expression was downregulated in both the 2D and 3D models by DHT, while the expression levels of *DKK1*, *IL-6*, *TGF*-β*1*, *PGD2*, and *CXXC5* were upregulated in both models by DHT.

**FIGURE 7:**
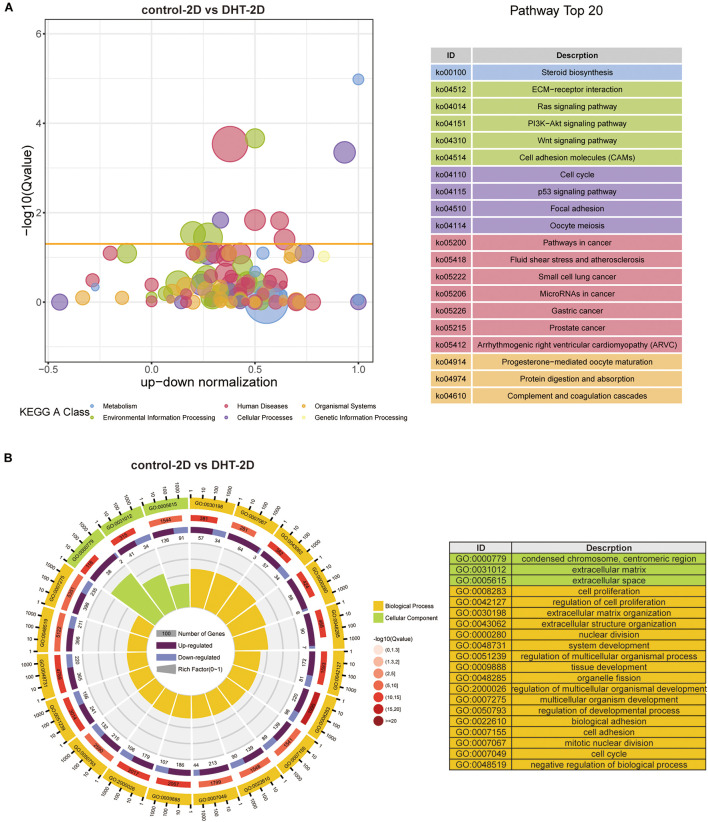
Kyoto Encyclopedia of Genes and Genomes and GO analysis of differentially expressed genes in 2D cultured dermal papilla cells. **(A)** Blue and red circles indicate metabolism and human diseases, respectively. The green circle indicates environmental information processing, whereas the brown circle represents organismal systems. The purple and yellow circles indicate cellular processes and genetic information processing, respectively. The size of the bubble represents the rich factor (gene number/total gene number), the *y*-axis represents –log10(*Q*-value), and the *x*-axis represents the normalized number of upregulated genes minus downregulated genes. **(B)** GO analysis of DEGs in 2D. Yellow represents biological process, whereas green represents cellular components. From the exterior to interior; the first lap indicates the top 20 GO terms and the coordinate ruler with the number of genes is presented outside the lap; the second lap indicates the total number of genes in the GO term and –log10(*Q*-value), wherein the greater number of genes, the longer the strip, and the larger the –log10(*Q*-value), the darker the color shade; the third lap represents the upregulation/downregulation gene ratio, wherein dark purple represents the upregulated gene proportion, light purple represents the downregulated gene proportion, and the specific value is shown below; and the fourth lap represents the rich factor of each term (number of genes/total number of genes), and each small cell of the background auxiliary line represents 0.1. The chart on the right shows the gene symbols corresponding to the top 20 GO IDs. DEGs, differentially expressed genes.

**FIGURE 8:**
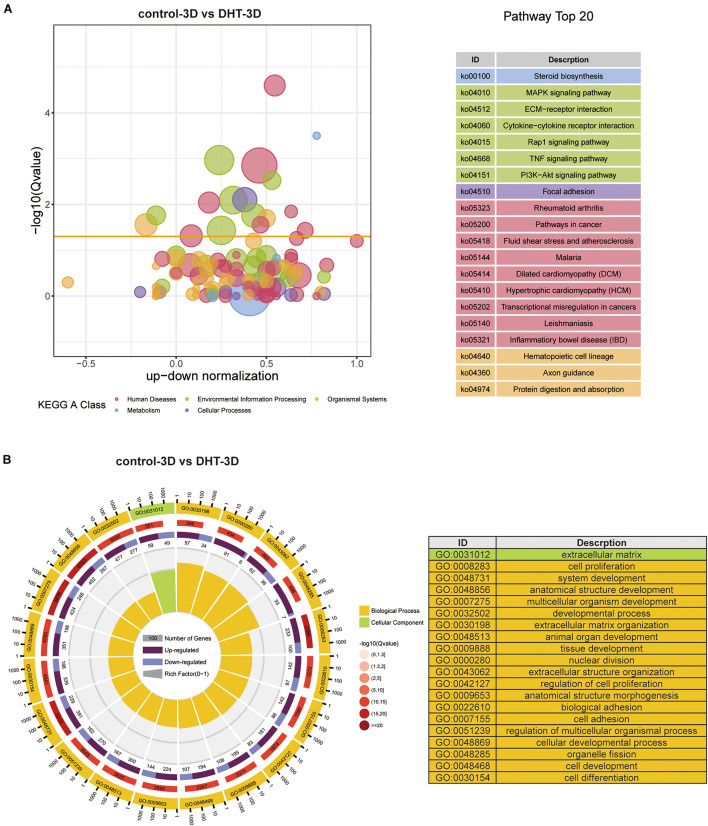
Kyoto Encyclopedia of Genes and Genomes and GO analysis of differentially expressed genes in 3D cultured dermal papilla cells. **(A)** Blue and red circles indicate metabolism and human diseases, respectively. The green circle indicates environmental information processing, whereas the brown circle represents organismal systems. The purple and yellow circles indicate cellular processes and genetic information processing, respectively. The size of the bubble represents the rich factor (gene number/total gene number), the *y*-axis represents –log10(*Q*-value), and the *x*-axis represents the normalized number of upregulated genes minus downregulated genes. **(B)** GO analysis of DEGs in 3D. Yellow: biological process, green: cellular component. From exterior to interior: first layer indicates the top 20 GO terms with the coordinate ruler indicating the number of genes presented external to the circle. Second layer (red): total number of genes in the GO term and –log10(*Q*-value), wherein a higher number of genes corresponds to a longer section, larger –log10(*Q*-value), and darker shade of red. Third layer: upregulation/downregulation gene ratio bar chart, wherein dark purple represents the upregulated gene proportion, light purple represents the downregulated gene proportion, and the specific value is shown below. Fourth layer: enrichment factor for each term (number of genes/total number of genes), with each small cell of the background auxiliary line representing 0.1. The chart on the right shows the gene symbols corresponding to the top 20 GO IDs. DEGs, differentially expressed genes.

Gene Ontology functional annotation and KEGG pathway analysis of the DEGs in the control-2D vs. DHT-2D and control-3D vs. DHT-3D (1,891 and 1,440 genes) groups were performed using the DAVID online tool. KEGG analysis revealed that alterations in metabolism were significantly altered during steroid biosynthesis in DEGs identified in the comparison groups. Meanwhile, in the 2D group, DEGs enriched in environmental information processing were primarily activated in the ECM–receptor interaction, Ras signaling pathway, PI3K-Akt signaling pathway, Wnt signaling pathway, and cell adhesion molecules (Figure 7A). The MAPK signaling pathway, TNF signaling pathway, ECM–receptor interaction, cytokine–cytokine receptor interaction, Rap1 signaling pathway, and PI3K–Akt signaling pathway were enriched in the 3D group (Figure 8A). Changes in the cellular processes of DEGs in the two comparison groups were related to focal adhesion in both groups. Additionally, DEGs in the 2D group were related to cell cycle, p53 signaling pathway, and oocyte meiosis. The KEGG network (Supplementary Figure 1) showed 10 nodes and 14 edges (black lines) in 3D groups, and the possible core pathways were the MAPK signaling pathway (six edges) and PI3K–Akt signaling pathway (five edges). However, fewer interactions between enriched pathways were established in the 2D network, with only four nodes and one edge. Based on the node size, the pathways enriched in the 2D model contained fewer genes than that in the 3D model. GO analysis revealed that DEGs in the 2D group were mainly enriched in condensed chromosomes, centromeric regions, ECMs, extracellular spaces in cell components (CC) and ECM organization, mitotic nuclear division, and extracellular structure organization in biological processes (BP) (Figure 7B). In the 3D group, changes in CC of DEGs were mainly enriched in the ECM, and changes in BP of DEGs were significantly enriched in ECM organization, nuclear division, extracellular structure organization, and organelle fission (Figure 8B).

### Gene Set Enrichment Analysis of the Gene Expression Files in Dihydrotestosterone-3D vs. Control-3D and Dihydrotestosterone-2D vs. Control-2D

**FIGURE 9:**
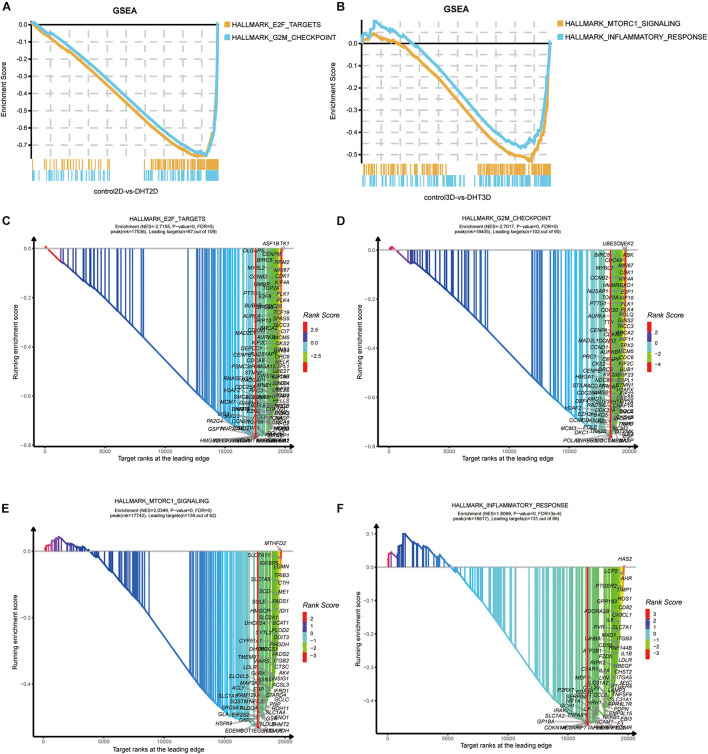
Gene Set Enrichment Analysis of all genes. The overall results of GSEA for 2D and 3D groups are depicted in **(A,B)**, respectively. Each plot shows the running ESs (*y*-axes) and the position of the members of each gene set in the ranked list of genes between control-3D and DHT-3D or control-2D and DHT-3D (*x*-axes). Each vertical bar corresponds to one gene under the gene set, and the peak represents the ES of this gene set. GSEA results of four gene sets by xGSEAdotplot function: E2F targets **(C)** and G2M checkpoint **(D)** in 2D, and mTORC1 signaling **(E)** and inflammatory response **(F)** in 3D. The title of the plot has two rows, wherein the first row is the name of the dataset, and the second row represents the ES information. The peak (red vertical bar) divides the curve into two sides; the right side is called the leading edge and the genes on the surface are called leading targets (candidate genes). The *x*-axis represents target ranks and the *y*-axis represents ES. DHT, dihydrotestosterone; ES, enrichment score; GSEA, Gene Set Enrichment Analysis.

As KEGG and GO analyses are mainly dependent on DEGs and ignore genes that do not meet the threshold, we performed GSEA to examine the functional analysis of all of the genes identified in this study. Based on the results of GSEA, two new significant gene sets were identified in the 2D and 3D groups, which were not identified by KEGG and GO analyses. Overall, G2M checkpoint and E2F targets and mTORC1 signaling and inflammatory response were significantly enriched in the 2D and 3D groups, respectively. GSEA was employed to identify the enriched gene sets between the control-3D and DHT-3D, including two datasets. G2M checkpoints [normalized enrichment score (NES) = −2.7017, *p* = 0, FDR = 0], E2F targets (NES = −2.7155, *p* = 0, FDR = 0) (Figures 9A,C,D), mTORC1 signaling (NES = 2.0349, *p* = 0, FDR = 0), and inflammatory responses (NES = 1.8069, *p* = 0, FDR = 0.0003) were enriched in the 3D group (Figures 9B,E,F).

### Identification of Overlapping Differentially Expressed Genes Between Dihydrotestosterone-3D vs. Control-3D and Dihydrotestosterone-2D vs. Control-2D

**FIGURE 10:**
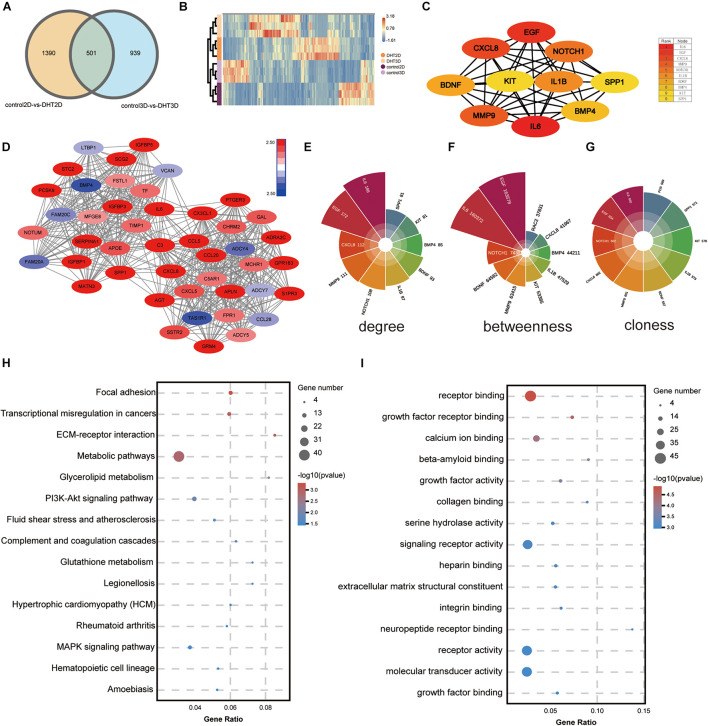
Identification of the most significant module and hub genes of overlapping genes. **(A)** Venn diagram illustrating the number of overlapping DEGs between the two comparison groups (2D vs. 3D); 501 DEGs were common to both groups. **(B)** Unsupervised hierarchical clustering of the 501 overlapped genes, computed by Euclidean distance clustering and illustrated using a heatmap with normalized raw *z*-scores. **(C)** PPI network of hub genes, with 10 nodes and 39 edges. The color depth of the nodes represents the degree level; the deeper the color, the higher the degree. **(D)** The most significant module was obtained from the PPI network with 45 nodes and 540 edges. The more the genes were downregulated, the darker the blue; the more the genes were upregulated, the darker the red. Top 10 genes in the network were ranked based on degree **(E)**, betweenness **(F)**, and closeness **(G),** and depicted using Polar histograms. Different colors represent different genes, and the larger the area of the fan, the larger the data, and vice versa. **(H)** KEGG analysis of overlapping genes. The size of the bubble represents gene number, the color depth represents the *Q*-value, and the rich ratio represents the gene number/total gene number on the *y*-axis. **(I)** GO analysis of overlapping genes: the size of the bubble represents the gene number, the color depth represents the *p*-value, and the rich ratio indicates the gene number/total gene number on the *y*-axis. DEGs, differentially expressed genes; PPI, protein–protein interactions.

After DEGs in the two comparison groups (1,891 in DHT-2D vs. control-2D and 1,440 in DHT-3D vs. control-3D) were identified, a Venn diagram (Figure 10A) was constructed to identify overlapping DEGs between the two comparison groups. Overall, 501 DEGs were common to both groups. Next, the DEGs were subjected to unsupervised hierarchical clustering and illustrated using an expression heatmap (Figure 10B).

### Kyoto Encyclopedia of Genes and Genomes and Gene Ontology Enrichment Analysis of Overlapping Genes

Kyoto Encyclopedia of Genes and Genomes pathway analysis (Figure 10H) revealed that the overlapping DEGs were mainly enriched in focal adhesion, transcriptional misregulation in cancers, ECM–receptor interaction, and metabolic pathways. GO analysis (Figure 10I) showed that the DEGs were significantly enriched in molecular function, including receptor binding and growth factor receptor binding.

### Construction of a Protein–Protein Interaction Network and Module Analysis

**FIGURE 11:**
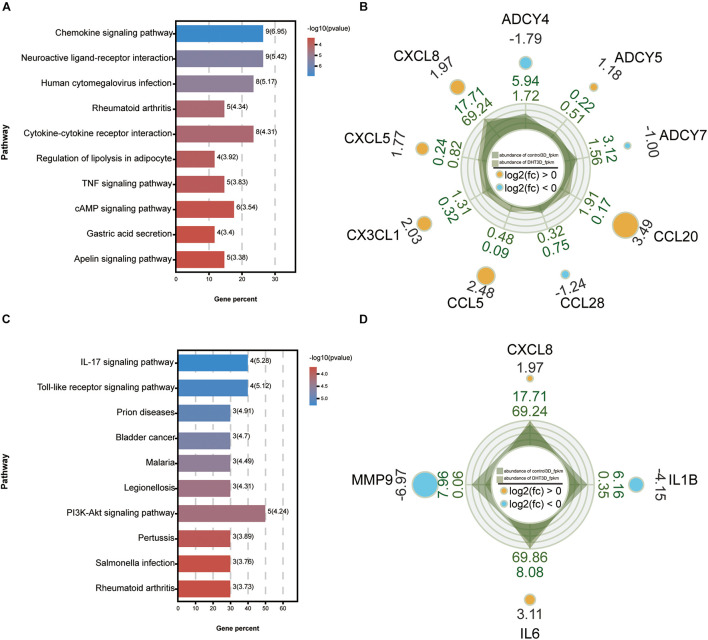
Kyoto Encyclopedia of Genes and Genomes analysis of the maximum related module and 10 hub genes. KEGG analysis of genes from the most significant module **(A)** and 10 hub genes **(C)**. Each column represents a pathway, and the height of the column represents the number of genes that pathway contains. Legends indicate –log10(*p*-value). The higher the value, the bluer the color, and the smaller the value, the redder the color. Rader plot of enriched genes in the chemokine signaling pathway from the most significant module **(B)** and four hub genes of IL-17 signaling pathway **(D)** in control-3D vs. DHT-3D. The shadow part is the line chart of the expression abundance [log10(RPKM)] of each gene in the two groups (dark green is control-3D and light green is DHT-3D). The values outside the shadow are the expression abundance (RPKM) of each group. The outermost value represents log2(FC); the circle size represents the multiple of the difference of a single gene between the two groups, the bigger the circle, the greater the value. The color indicates upregulated and downregulated genes: orange indicates upregulation [log2(FC) > 0] and blue indicates downregulation [log2(FC) < 0], and the corresponding number is log2(FC). DHT, dihydrotestosterone; FC, fold change; RPKM, reads per kilo base per million mapped reads.

A PPI network of the DEGs was constructed using Cytoscape, and the most significant module, with 45 nodes and 540 edges, was obtained (Figure 10D) using the MCODE application. Then, GO and KEGG analyses of the DEGs, which included functional classification and enrichment analyses, were performed using the DAVID online tool. The results showed that the chemokine signaling pathway was significantly enriched by nine DEGs, including *CXCL8*, *CXCL5*, *CX3CL1*, *CCL5*, *CCL28*, *CCL20*, *ADCY7*, *ADCY5*, and *ADCY4* (Figures 11A,B).

### Selection and Analysis of Hub Genes

The top 10 genes with degrees ≥10 were identified as hub genes. The PPI network of the 10 hub genes (*IL-6*, *EGF*, *CXCL-8*, *MMP9*, *NOTCH1*, *IL-1*β, *BDNF*, *BMP4*, *KIT*, and *SPP1*) along with 10 nodes and 39 edges were identified using the Cytoscape CytoHubba application (Figure 10C). Moreover, we applied two algorithms (“betweenness” and “closeness”) to further verify the importance of the 10 hub genes. The results showed that the node scores were basically consistent, and nine genes were identified by the three algorithms except *SPP1*, suggesting that the hub genes were indeed important (Figures 10E–G). KEGG analysis of these 10 hub genes revealed that four of them (*MMP9*, *CXCL-8*, *IL-1*β, and *IL-6*) were enriched in the IL-17 signaling pathway (Figures 11C,D). To analyze the function and interaction of these genes, GO_FULL analysis was used to calculate overrepresented GO terms in the network and displayed them as a network of significant GO terms using the Cytoscape BiNGO application. The biological process and molecular function analysis of the hub genes is shown in Supplementary Figure 2. According to BiNGO analysis, the top 10 GO terms the hub genes were involved in were primarily related to cell chemotaxis and regulation of chemokine biosynthetic, indicating that DHT may induce DPCs to secrete related chemokines and produce cell chemotaxis.

### Expression Profiles of the Hub Genes and Quantitative Real-Time Reverse Transcription Polymerase Chain Reaction Verification

**FIGURE 12:**
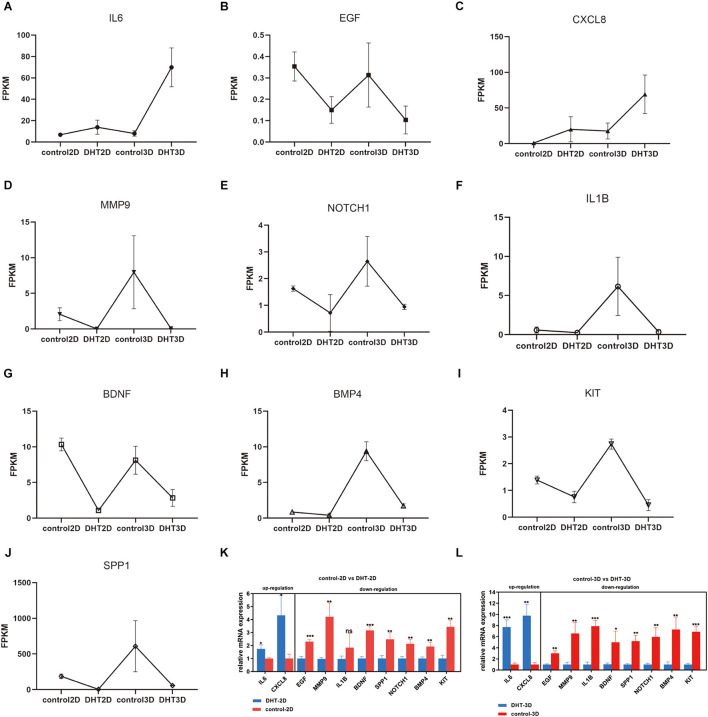
Expression of hub genes and qRT-PCR verification. **(A–J)** Expression of 10 hub genes in four groups. *y*-axis item is FPKM. Relative mRNA expression levels of two upregulated and eight downregulated hub genes in the 2D **(K)** and 3D **(L)** groups. ns, not significant *p < 0.05; **p < 0.01; ***p < 0.001 when compared with the respective controls by one-way ANOVA and Student’s *t*-test. FPKM, fragments per kilo base per million mapped reads.

Control-2D, DHT-2D, control-3D, and DHT-3D fragments per kilobase million values of the hub genes are depicted in Figures 12A–J, revealing the changing trends of the hub genes among the four groups. The expression of *IL6* and *CXCL8* increased with an increase in DHT concentration in both the 2D and 3D groups, while the expression levels of *EGF*, *MMP9*, *IL1B*, *KIT*, *SPP1*, *BMP4*, and *NOTCH1* exhibited the opposite trend. The mRNA expression levels of the 10 hub genes were examined using qRT-PCR, and this confirmed the results of RNA-seq (Figures 12K,L).

### Interactions of Transcription Factor-Gene and MicroRNA With Hub Genes

**FIGURE 13:**
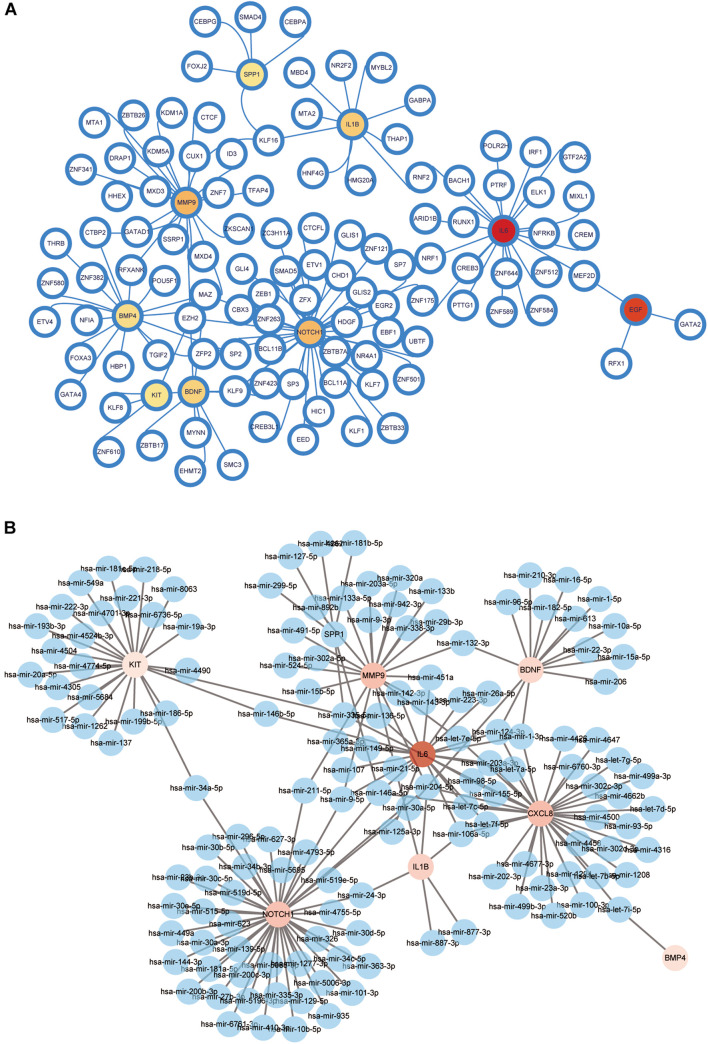
Network for transcription factor-gene and miRNA interaction with hub genes. **(A)** Network for TF-gene interaction with hub genes. The white node represents the TF-genes and other nodes represent hub genes. The network consists of 116 nodes and 126 edges. **(B)** Network for miRNA interaction with hub genes. The blue nodes represent the miRNA genes, and other nodes represent hub genes. The network consists of 151 nodes and 176 edges. TF, transcription factor.

Transcription factor-gene interactions were collected using NetworkAnalyst. The TF-genes and miRNA for the hub genes were identified. The interaction of TF regulators and miRNAs with hub genes was visualized in Figures 13A,B.

### Transcription Factor–MicroRNA Coregulatory Network

A TF–miRNA coregulatory network was generated using NetworkAnalyst. The analysis of the TF–miRNA coregulatory network delivered miRNAs and TFs interaction with the hub genes. This interaction may explain the regulation of expression of overlapping genes. The network created for the TF–miRNA coregulatory network comprises of 226 nodes and 364 edges; There were 148 miRNAs and 107 TF-genes that interacted with the hub genes. Supplementary Figure 3 displays the TF–miRNA coregulatory network.

## Discussion

Some studies have reported that the full dermal papilla transcriptional signature can be partially restored by the growth of papilla cells in 3D spheroid cultures (Higgins et al., 2013; Lin G. et al., 2020). Thus, 3D DPC cultures represent useful models of dermal papillae. In the present study, we compared the effectiveness of 2D- and 3D-cultured DPCs in identifying new key genes and pathways associated with AGA under DHT treatment using high-throughput RNA-seq technology.

Androgen receptors are localized in the dermal papillae of hair follicles, making the dermal papillae the target of androgen action. Significantly more ARs exist in the DPCs of balding scalp hair follicles than in those derived from non-balding follicles (Hibberts et al., 1998). AR methylation, which results in decreased AR expression, may prevent the miniaturization of hair follicles and hair loss (Cobb et al., 2011). Therefore, DPCs in culture models should be rich in ARs to further simulate androgen-induced hair loss *in vivo*. Accordingly, we selected DPCs with the highest AR expression (P2) for the 2D and 3D co-culture experiment. The transport of ARs from the cytoplasm to the nucleus also indicated activation of the androgen metabolic pathway, which interacts with pathways related to hair follicle growth, such as the Wnt/β-catenin pathway (Chesire and Isaacs, 2002; Kitagawa et al., 2009).

Herein, we used cell models for the co-culture experiment instead of animal models and AGA hair follicles because human hair DPCs were the focus of the study. Over the past 30 years, DPCs have been clearly shown to play indispensable roles in the induction and maintenance of epithelial cell growth (Reynolds et al., 1991), as well as in the release of growth factors that act in a paracrine manner on other follicle cells (Paus and Cotsarelis, 1999). Another reason is that animal and human hair follicles consist of several types of cells, such as dermal fibroblasts, epidermal keratinocytes, hair matrix cells, inner root sheath cells, and ORS cells (Shimomura and Christiano, 2010), which may affect the cell of interest. Moreover, there are some limitations associated with animal models, such as species differences and poor ability to predict the actual human response. Additionally, once the organs or tissues of interest are isolated, it becomes impossible for them to be stimulated by androgen, making it difficult to explore the paracrine effect of a single cell type (DPC) on hair follicle epidermal cell growth under androgen treatment. In previous studies, researchers tended to choose a group of factors and a signal pathway, often leading to incomplete results. Therefore, we used specific cell models to identify genes related to AGA under DHT treatment.

In our models, DPCs from balding men were treated with different concentrations of DHT and ORS cell proliferation was used to represent hair follicle growth *in vivo*, with an increase in ORS number indicating hair follicle growth. A concentration of only 10 μM DHT significantly inhibited the proliferation of both 2D- and 3D-cultured DPCs. The ORS cell number in the control-3D group was significantly higher than that in the control-2D group; however, DHT treatment significantly decreased the ORS cell number in both models. Moreover, DHT treatment did not affect the proliferation of epidermal cells without DPCs. This confirmed that 3D-cultured DPCs induce the proliferation of epidermal cells and that the effect of DHT on the hair follicle epidermis is mediated by DPCs indirectly, which is consistent with the findings of previous *in vivo* studies (Pan et al., 1999; Kwack et al., 2008). Additionally, the significant decrease and increase in Ki67 and cleaved caspase-3 expression in ORS cells in both models indicates a decrease in ORS cell proliferation, which may be mediated by DPC-secreted proteins under DHT treatment. Overall, the data indicate that our co-culture system mimicked androgen-induced alopecia to some extent.

In this study, we pioneered the use of ultra-low attachment 96-well plates to convert scattered 2D structured DPCs into 3D structured multicellular spheres with a diameter of 200 μm, similar to the structure and size of dermal papillae of hair follicles *in vivo*. Generally, 2D cells do not have the same architecture as cells *in vivo* that are arranged in 3D structures unattached to planar surfaces (Haykal et al., 2020), and cells in monolayer culture proliferate at an unusually faster rate than cells *in vivo* (Langhans, 2018). Indeed, we confirmed a higher proliferation rate of DPCs in the 2D model than in the 3D model. Moreover, 2D cells differ in gene and protein expression and dynamic processes, such as cell migration and division compared with that of vivo models (Ben-David et al., 2017); this is because the connection between cells and the ECM are not recapitulated in 2D culture models. Herein, RNA-seq of DPCs used to identify previously unknown transcripts (Mortazavi et al., 2008) demonstrated that DEGs between the models were mainly enriched in ECM–receptor interaction and that genes related to ECM–receptor interaction were upregulated due to cell aggregation. This indicates that compared to the ECM in the 2D model, the ECM in the 3D model was more similar to the dermal papillae of hair follicles. Additionally, 3D-cultured DPCs possessed high hair follicle regeneration ability (upregulated β−catenin and NCAM genes), similar to that of primary DPCs *in vivo* (Lin G. et al., 2020), indicating that the 3D model is more representative of the hair follicle microenvironment (Lin B. et al., 2020). β−Catenin and NCAM are dermal papillae biomarkers (Müller-Röver et al., 1998; Enshell-Seijffers et al., 2010), and β−catenin promotes hair follicle regeneration and the expression of dermal papillae-specific ECM *in vivo* (Maretto et al., 2003). Therefore, the advantages of the 3D model over the 2D model indicates that there may be differences in the DPC response to androgen. Most previous studies examined the pathogenesis of AGA using 2D co-culture models; however, as 2D models are not a proper representation of *in vivo* conditions, important key genes may not have been identified.

The KEGG network diagram demonstrated that the 3D model had more nodes (pathways), genes, and complex relationships than the 2D model, suggesting that the 3D model provides a more accurate representation of *in vivo* conditions. DEGs in 3D-cultured DPCs were primarily enriched in the MAPK signaling pathway, TNF signaling pathway, ECM–receptor interaction, cytokine–cytokine receptor interaction, Rap1 signaling pathway, PI3K–Akt, and extracellular matrix organization in our study. From these results, the most widely studied pathway related to AGA is the Wnt signaling pathway, which involves functional crosstalk between AR and Wnt signaling pathways in target tissues (Van Mater et al., 2003; Lo Celso et al., 2004; Kitagawa et al., 2009). Hair follicle regeneration is reportedly related to upregulation of the Akt pathway, which is induced by HFSC proliferation (Kim et al., 2019) and downregulation of the p53 pathway (Qu et al., 2021). Activated platelet-rich plasma supernatant also increases the quantity of DPCs by activating the MAPK and Akt signaling pathways (Xiao et al., 2019). Meanwhile, a significant link exists between TNF-α antagonist exposure and the development of alopecia (Béné et al., 2014) and EGFR–RAS–RAF signaling in epidermal stem cells is inextricably linked to the beginning and end of the hair cycle (Doma et al., 2013). Moreover, DPC proliferation is promoted by VEGF through the VEGFR-2/ERK pathway (Qu et al., 2021). Therefore, the pathways observed to be enriched in the current study may be related to the effect of DPCs on epidermal composition under DHT treatment.

Gene Set Enrichment Analysis identified two gene sets in the 3D (mTORC1 signaling and inflammatory response) and 2D (E2F targets and G2M checkpoint) models. mTOR was previously reported to activate HFSCs (Castilho et al., 2009) and DHT to activate mTOR, which inhibits DPC proliferation (Kang et al., 2015). mTORC1 signaling also inhibits BMP signaling, which may be the main mechanism facilitating HFSC activation (Deng et al., 2015). Chai et al. (2019) discovered that autophagy through inhibition of mTOR signaling is sufficient for activating telogen, initiating a new anagen phase of hair growth. In the past, microinflammation of hair follicles has been recognized to play an active role in the progression of AGA (Mahé et al., 2000). Similarly, hair follicle microinflammation appeared necessary to AGA, and collagen gradually accumulated in the perifollicular sheath, leading to fibrosis or complete destruction of hair follicles (El-Domyati et al., 2009). E2F, a TF encoding cell cycle genes, is primarily involved in DNA replication and cell cycle progression (Bracken et al., 2004), whereas G2M represents genes involved in the G2/M checkpoint of the cell cycle. DHT reduces cyclin D1, CDK2, and p27^*kip*1^ levels in immortalized DPCs, arresting the cell cycle from the G1 phase to the S phase (Kang et al., 2015). However, these previous findings showed that the above-mentioned pathways are mainly associated with hair growth and hair cycle regulation, with limited studies on their role in androgen-induced alopecia.

Protein–protein interaction analysis of the overlapping genes showed that the chemokine signaling pathway was significantly enriched under DHT treatment. However, few reports have suggested that chemokines are associated with DPCs under DHT treatment and can cause androgen-induced alopecia. A chemokine is a small chemotactic cytokine and is known for inducing leukocyte migration to target tissue in the inflammatory response. However, chemokines also play important roles in the development and homeostasis of hematopoietic and immune cells (Rossi and Zlotnik, 2000). Lefebvre et al. (2012) proposed an interaction between chemokines and skin appendage morphogenesis and reported that the number of primary hair follicles was reduced in *CXCR3*-deficient mice. Heath and Mueller (2012) reported that some chemokines that affect the recruitment of Langerhans cells to the epidermis are expressed in specific areas of hair follicles. Based on our results, we speculated that chemokine expression in DPCs is site-specific. The expression of chemokines under DHT treatment resulted in changes in cell migration, cell survival, and adhesion.

The identified top 10 hub genes showed a significant increase in *IL6* and *CXCL8* expression in the absence of DHT, and a significant decrease in *EGF*, *MMP9*, *NOTCH1*, *IL-1*β, *BDNF*, *BMP4*, *KIT*, and *SPP1* expression in the presence of DHT. However, the specific effect of chemokines needs to be further studied. Epidermal growth factor (EGF) has been identified in follicular differentiation (du Cros, 1993) and promotion of the transition of the hair cycle from telogen to anagen, as well as growth of the hair shaft (Zhao et al., 2020). Rendl et al. (2008) found that when DPCs are cultured in a medium containing BMP4 *in vitro*, there is a significant increase in hair follicle formation, hair growth, and alkaline phosphatase expression. Notch1 deletion inhibits the maturation of hair follicles from the late embryonic stage (Blanpain et al., 2006; Moriyama et al., 2008); one of the ways β-catenin regulates its effects on the hair follicle cycle and maintenance is through the Notch pathway (Vauclair et al., 2005; Estrach et al., 2006). A recent study showed that skin bacteria promote the complete regeneration of skin and hair follicles in wounds through the IL-1β pathway (Wang et al., 2021). However, most of these factors have not been revealed in the pathogenesis of AGA and the other five genes have not yet been studied.

A TF-gene and miRNA interaction network was obtained with the hub genes. TF-genes are involved in the regulation of gene expression through TF binding with targeted genes and miRNAs; they can also regulate gene expression through mRNA degradation (Wang et al., 2018). From the network, NOTCH1 showed a high interaction rate with other TF-genes and miRNAs. The degree value of NOTCH1 was 37 in the TF-gene interaction network and 44 in the miRNA interaction network. Among all TF-genes and miRNAs, NFKB1 had the highest degree value of 7. EZH2, MAZ, has-miR-204-5p, and has-miR-146a-5p exhibited significant interaction with degree values of 5, 4, 5, and 4, respectively, in the TF-gene and miRNA interactions network. EZH2 accelerates differentiation of HFSCs and hair growth by downregulating miR-22 (Cai et al., 2020). miR-200 family members play a vital role in the regulation of cell adhesion and proliferation in hair morphogenesis (Hoefert et al., 2018). Given that regulatory biomolecules are potential biomarkers in numerous complex diseases, further studies are needed to examine the roles of the hub genes and chemokines on morphological changes of DPCs and animal hair follicles *in vitro* and *in vivo*.

The two culture models employed in this study were sufficient to identify the effects of DHT-treated DPCs on hair follicle keratinocytes to a limited extent. We identified 501 overlapping DEGs, 10 hub genes, chemokines, and related signaling pathways in DPCs cultured using the 2D and 3D models. The identified DEGs were involved in AGA in DPCs and may be potential biomarkers for hair growth. The hub genes may also be potential biomarkers for the diagnosis and treatment of androgen-induced alopecia. Additionally, our findings suggest that chemokines and the IL-17 pathway may be related to AGA; however, this requires further investigation. Moreover, upregulation of *EGF*, *MMP9*, *NOTCH1*, *IL-1*β, *BDNF*, *BMP4*, *KIT*, and *SPP1*, and downregulation of *IL6* and *CXCL8* may lead to the growth and maintenance of hair follicles. Overall, the findings provide the first global transcriptome of DPCs in 2D and 3D co-cultured models under DHT treatment and highlight the 3D model as a useful tool for elucidating hair follicle induction and hair growth.

## Data Availability Statement

The data presented in the study are deposited in the GEO repository, accession number GSE178374.

## Ethics Statement

The studies involving human participants were reviewed and approved by the Medical Ethical Committee of the Southern Medical University. The patients/participants provided their written informed consent to participate in this study.

## Author Contributions

YZ, JH, DF, ZL, and HW performed all of the experiments and prepared the figures and schemes. JW, QQ, KL, and ZF provided statistical assistance. YZ wrote the first draft of this manuscript. JH and YM revised the manuscript for important intellectual content. ZH and JH contributed to the conception and design of the study. All authors contributed to manuscript revision, and read and approved the submitted version.

## Conflict of Interest

The authors declare that the research was conducted in the absence of any commercial or financial relationships that could be construed as a potential conflict of interest.

## Publisher’s Note

All claims expressed in this article are solely those of the authors and do not necessarily represent those of their affiliated organizations, or those of the publisher, the editors and the reviewers. Any product that may be evaluated in this article, or claim that may be made by its manufacturer, is not guaranteed or endorsed by the publisher.

## Footnotes

1
http://www.r-project.org/

2
https://www.omicshare.com

3
https://www.kegg.jp/

4
http://david.ncifcrf.gov

5
http://software.broadinstitute.org/gsea/index.jsp

6 http://string-db.org; version 10.5

7
https://www.networkanalyst.ca/
