# Supplementary material for: Transcriptome Analysis Reveals an Inhibitory Effect of Dihydrotestosterone-Treated 2D- and 3D-Cultured Dermal Papilla Cells on Hair Follicle Growth
Source: Front Cell Dev Biol. 2021 Sep 17;9:724310. doi: 10.3389/fcell.2021.724310 (PMC8484716; doi:10.3389/fcell.2021.724310)
Supplement: Supplementary file 1 [file Data_Sheet_1.pdf]

frontiers

Supplementary Material

1.1 Supplementary Figures

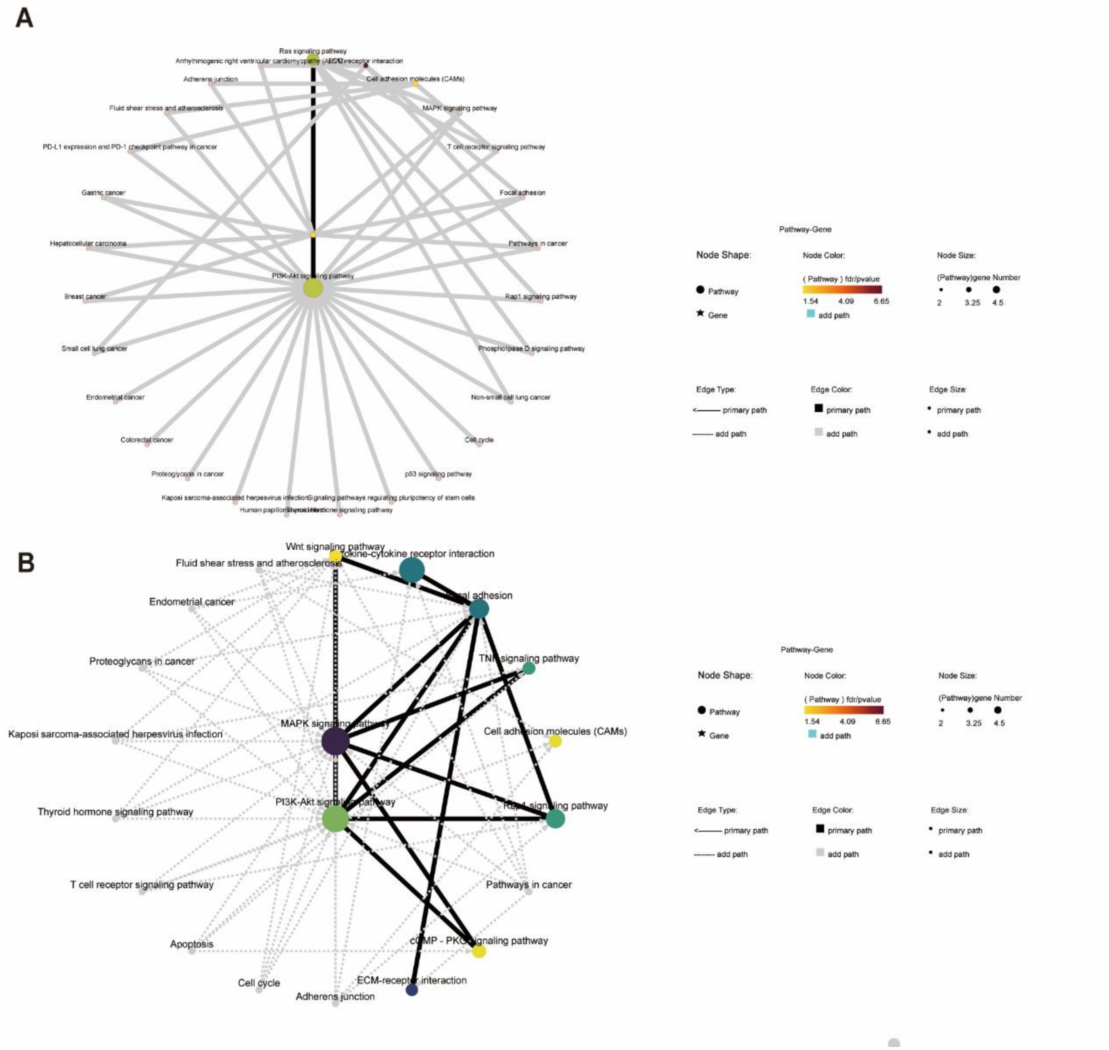

Supplementary Figure 1. KEGG network of DEGs identified in the 2D (A) and 3D (B) groups. Each node represents a KEGG pathway; the size of the node represents the number of genes enriched in the pathway; the color of the node indicates the *p*-value, and a grey node represents the supplementary adjacent pathway from the KEGG connection database. A solid line in the figure indicates that there is a connection between the enriched pathways; a dotted line indicates the connection between the supplementary pathway and the enriched pathway.

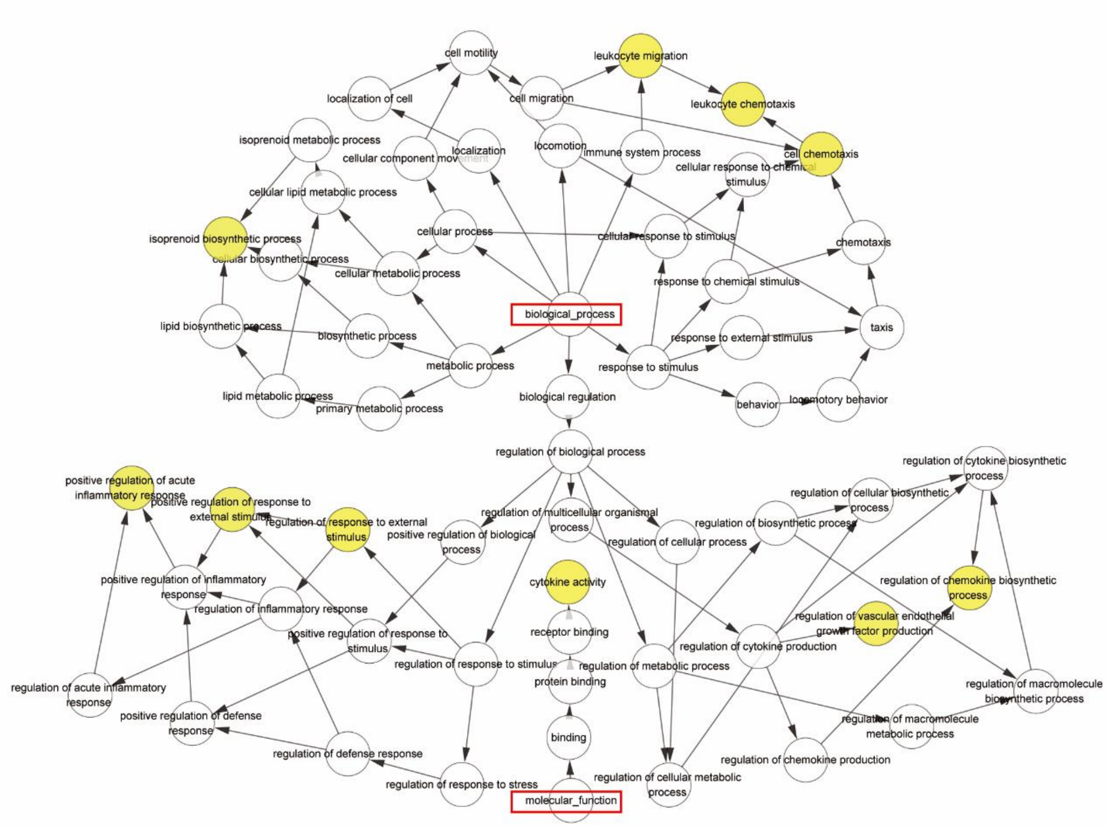

Supplementary Figure 2. GO_FULL analysis of 10 hub genes. GO_FULL analysis, including biological process, molecular function, and cellular component analyses of hub genes, were performed using BiNGO. The yellow nodes represent the top 10 terms having the highest corrected *p*-values of ontologies. $p < 0.01$ was considered statistically significant.

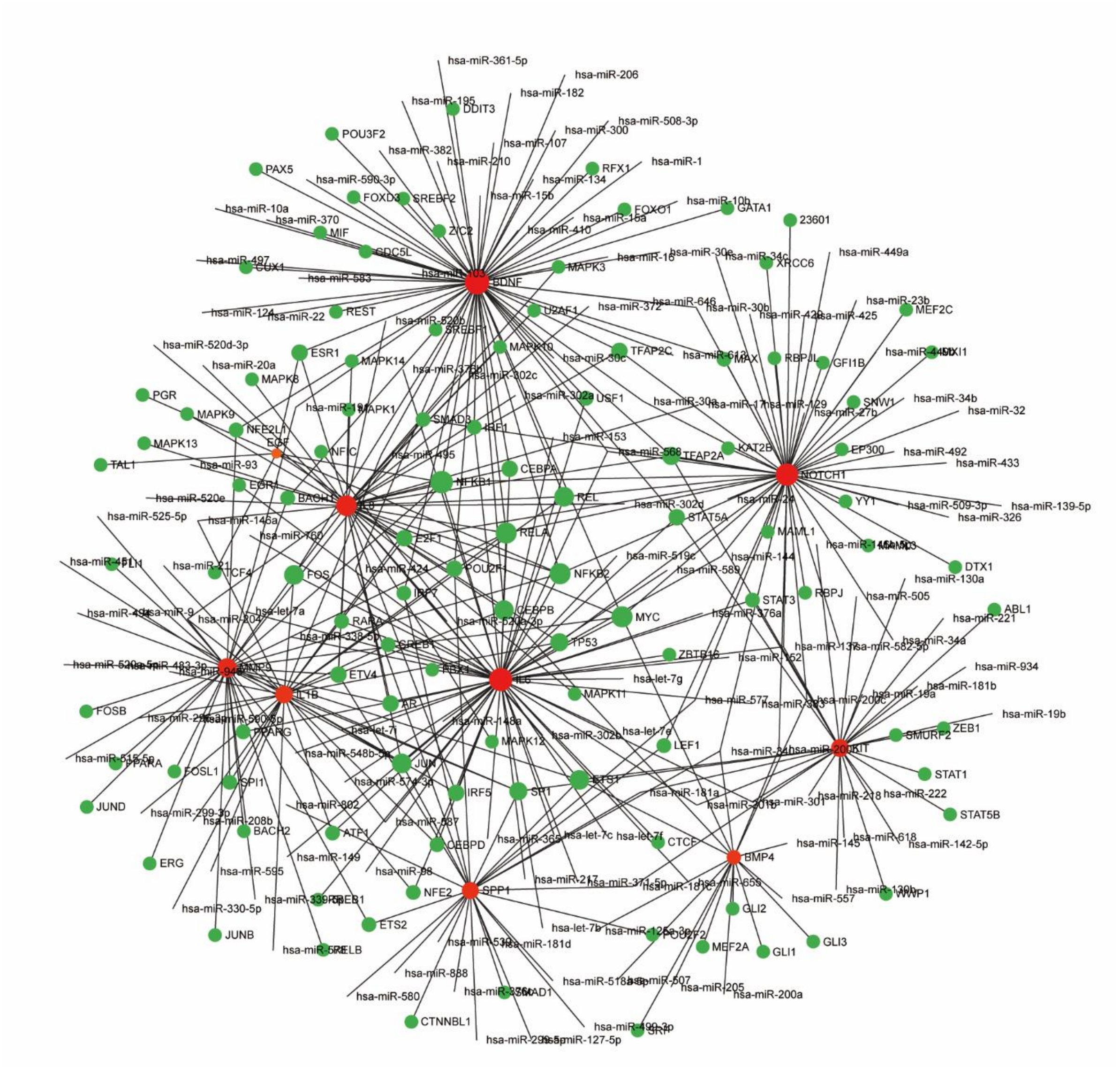

Supplementary Figure 3. TF-miRNA coregulatory network. The network consists of 266 nodes and 364 edges including 148 TF-genes, 107 miRNAs, and 9 hub genes. The nodes in red are the hub genes, a green node represents TF-genes, and other nodes indicate miRNAs.
